# Jumeaux conjoints au niveau d’une omphalocèle commune avec extrophie cloacale et ambigüité sexuelle

**DOI:** 10.11604/pamj.2014.17.243.2418

**Published:** 2014-03-31

**Authors:** Majdouline Boujoual, Hamid Madani, Housain Benhaddou, Mohamed Belahcen

**Affiliations:** 1 Gynécologie Obstétrique, Faculté de Médecine et de Pharmacie Oujda, Université Mohamed I, Maroc; 2 Anesthésie Réanimation, Faculté de Médecine et de Pharmacie Oujda, Université Mohamed I, Maroc; 3 Chirurgie Pédiatrique, Faculté de Médecine et de Pharmacie Oujda, Université Mohamed I, Maroc

**Keywords:** Jumeaux conjoints, omphalocèle, extrophie cloacale, ambigüité sexuelle, conjoined twins, omphalocele, cloacal exstrophy, sexual ambiguity

## Abstract

Les jumeaux conjoints sont considérés comme étant une complication rare et grave des grossesses monozygotes. Le diagnostic anténatal permet de définir avec précision les structures communes, de rechercher une anomalie congénitale associée, d'organiser l´accouchement et la prise en charge néonatale. Nous présentons un cas rare de jumeaux conjoints dont la fusion se situait au niveau d'une omphalocèle commune associée à une extrophie cloacale, ambiguïté sexuelle et pieds bots. Le diagnostic a été méconnu pendant la grossesse, ce qui a engendré une dystocie lors de l'accouchement. L'issue a été fatale malgré une tentative de séparation et des mesures de réanimation. Ce cas illustre la difficulté liée d'une part à la méconnaissance du diagnostic, d'autre part au caractère urgent de la césarienne et de la séparation chirurgicale.

## Introduction

La prévalence des jumeaux conjoints est de 1/250.000 nouveaux nés. Environ 40 à 60% des cas sont morts nés et 35% survivent uniquement le premier jour [1]. C'est une complication rare et grave des grossesses monozygotes due au clivage tardif au stade de disque embryonnaire [2]. Le diagnostic anténatal permet de définir avec précision les structures et les rapports existant entre les deux jumeaux, de rechercher une anomalie congénitale associée, d'effectuer une surveillance mensuelle de cette grossesse, d'informer les parents, et d'organiser l´accouchement dans une maternité de niveau III [3]. Le pronostic dépend de la nature et l'extension des organes communs ainsi que l'association à d'autres malformations [4]. Nous présentons un cas rare de jumeaux conjoints dont la fusion se situait au niveau d'une omphalocèle commune, associée à une extrophie cloacale et ambiguïté sexuelle. Nous insisterons sur l´intérêt d´un diagnostic anténatal précoce afin d'éviter les accidents mécaniques d´un accouchement dystocique et de programmer une prise en charge néonatale multidisciplinaire.

## Patient et observation

**Figure 1:**
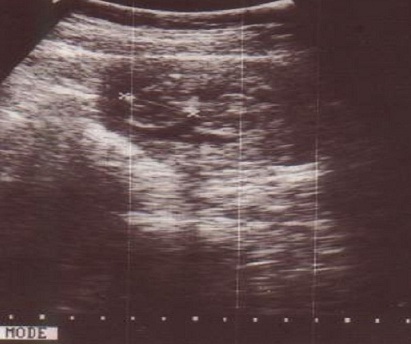
Echographie obstétricale des jumeaux conjoints à 13 semaines d’aménorhées

**Figure 2:**
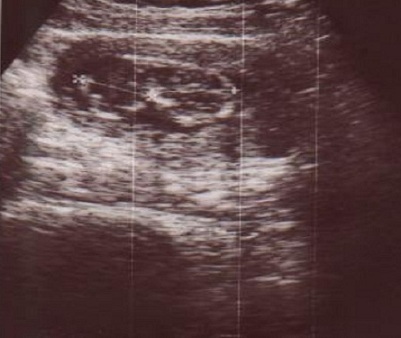
Echographie obstétricale des jumeaux conjoints à 13 semaines d’aménorhées

**Figure 3:**
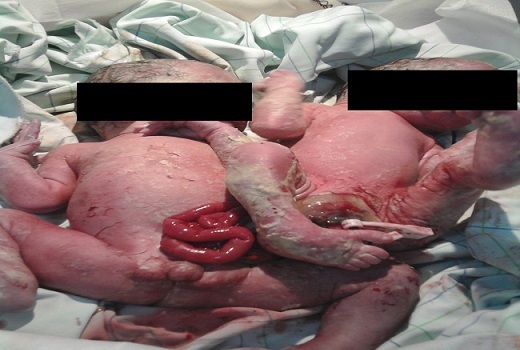
Jumeaux conjoints avec omphalocèle commune rompue, ambiguité sexuelle et pièds bots

**Figure 4:**
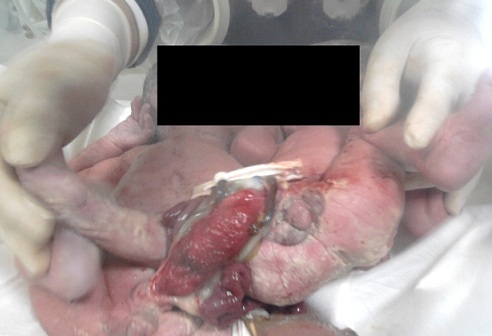
Jumeaux conjoints avec extrophie cloacale :omphalocèle, extrophie vésicale et impérforation anale

Parturiente âgée de 28 ans, primigeste primipare, ayant un antécédent d'infertilité de trois ans dans un contexte d'hyper prolactinémie traitée, chez qui le suivi prénatal avait diagnostiqué une grossesse gémellaire sans suspicion de jumeaux conjoints (Figure 1, Figure 2). La patiente est rentrée en travail à 38 semaines d'aménorrhées (SA), elle nous a été référée pour dystocie sur une grossesse gémellaire. A l'examen, on notait un syndrome de lutte avec extériorisation d'un seul cyanosé sans progression de la descente. Une césarienne a été indiquée en urgence, a permis l'extraction de deux jumeaux conjoints vivants accolés au niveau d'une Omphalocèle commune rompue. Ils présentaient des pieds bots, une ambigüité sexuelle avec un bourgeon génital médian type masculin, hypospadias postérieur et un scrotum vulviforme sans gonades palpables (Figure 3). De plus, il existait une disjonction pubienne associée à une extrophie vésicale et un périnée effacé sans anus (Figure 4).

**Figure 5:**
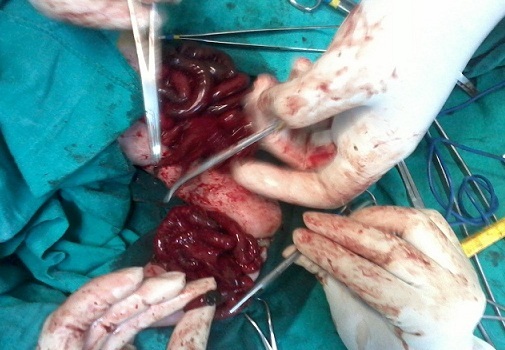
Intervention de séparation des jumeaux avec résection grélique en urgence du fait de l’ischémie intestinale et iléostomies

**Figure 6:**
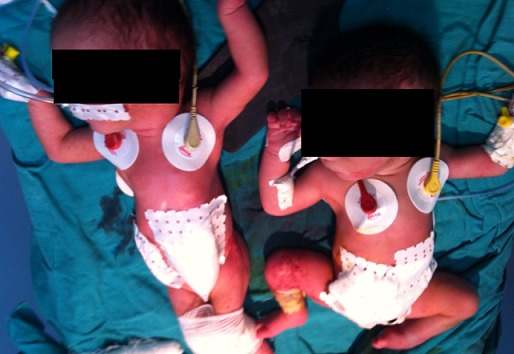
Jumeaux après la séparation chirurgicale

L'évolution a été marquée par la survenue d'une ischémie intestinale quelques heures après la naissance, on a posé alors l'indication de séparation chirurgicale urgente. L'exploration chirurgicale a montré que ces jumeaux étaient liés uniquement par l'intestin grêle avec à la partie moyenne un diverticule de Meckel. Le premier présentait un bout de colon atrétique et un cul de sac rectal derrière la vessie s'abouchant sur le tractus urogénital. Tandis que le deuxième, présentait la même disposition intestinale et urogénitale mais sans colon individualisable. Ils ont bénéficié d'une résection grélique segmentaire avec iléostomies et fermeture des plaques vésicales après rapprochement des hémi pubis (Figure 5). Ceci a été suffisant pour séparer les deux bébés (Figure 6), mais l'issue a été fatale marquée par le décès d'un jumeau le 1er jour, et de l'autre au 3ème jour.

## Discussion

La rareté des jumeaux conjoints est due à la rareté de la grossesse gémellaire, du monozygotisme, et de la division embryonnaire après le 14ème jour post fécondation ou de la fusion partielle des deux lignes primitives ce qui conduit aux monstres doubles [5]. Les jumeaux conjoints sont génétiquement identiques et ont le même sexe [1]. En effet, le sexe féminin est dominant dans 70 à 75% des cas, contrairement à notre cas où les jumeaux présentaient une ambigüité sexuelle [2]. En fonction du site d´union, de son importance, des organes communs et de la symétrie, une classification sémiologique a été établie : dans 75 % des cas, la fusion intéresse le thorax et/ou l´abdomen jusqu´à l´ombilic [6]. Le sous groupe des « jumeaux omphalopages minimement conjoints » présente une union de la paroi abdominale sous-ombilicale sans union du périnée ni du bassin osseux : il se caractérise par l'union des cavités péritonéales à travers un défaut de la paroi abdominale antéo-inférieure, de l´intestin grêle distal, de l'ouraque et l'association aux malformations ano-rectales (tableau I)' 14 cas ont été rapportés dans la littérature, ces observations suggèrent que le mécanisme pourrait être dû à une simple anomalie d'insertion du canal allantoïde entrainant un défaut de fermeture de la ligne médiane et altérant ainsi le développement normal du cloaque par traction vers le haut de la membrane cloacale [7]. Notre cas rapporté est rare puisqu'en plus de l'omphalocèle et l'extrophie cloacale, ces jumeaux présentaient une ambigüité sexuelle.

Le diagnostic de jumeaux conjoints est possible avant la 12^ème^ semaine d'aménorrhée grâce à l'échographie [5] qui montre la présence d'une seule vésicule vitelline extra-amniotique avec un embryon unique d'aspect bifide [8]. Une surveillance est nécessaire pour confirmer le diagnostic notamment devant l'indistinction des corps f'taux, l'absence de membrane séparatrice, la présence de plus de trois vaisseaux dans le cordon ombilical, la présence de têtes demeurant au même niveau et dans le même plan corporel, avec proximité inhabituelle des membres et incapacité de modifier les positions f'tales dans le temps [9]. De plus, elle permettra de rechercher une anomalie congénitale associée [3]. Enfin, les anomalies de « cohabitation» des 2 jumeaux comme les déformations des pieds ou les asymétries cranio-faciales peuvent être notées, souvent ces jumeaux partagent la même anomalie [10]. Toutefois, les jumeaux omphalopages minimement conjoints peuvent avoir une liaison souple qui leur permet de se déplacer de façon relativement autonome entrainant des difficultés diagnostiques à l´échographie prénatale [7], ceci concorde avec notre observation où le diagnostic n'a pas été fait en prénatal malgré la réalisation de plusieurs échographies obstétricales.

Une fois le diagnostic posé, la prise en charge obstétricale dépend de la décision conservatrice ou non. En cas de décision non conservatrice, est pratiquée une interruption de grossesse par voie basse avant 24SA, et par césarienne au-delà de 24 SA [2]. Lorsque le diagnostic est méconnu, l'accouchement ou la tentative d'accouchement par voie basse peut engendrer de graves lésions maternelles [5].

Une séparation programmée est recommandée, elle permet une évaluation détaillée de l´anatomie, diminue le risque relatif à l´anesthésie et permet de planifier des stratégies chirurgicales. Cependant, la séparation urgente peut être indiquée en cas de: mort-né, rupture de l'omphalocèle ou du laparoschisis, traumatisme des structures communes, occlusion intestinale, uropathie obstructive, insuffisance cardiaque congestive, gêne respiratoire et mise en jeu du pronostic vital d'un jumeaux par rapport à l´autre. De plus, ces jumeaux qui présentent des anomalies anorectales nécessitent souvent une dérivation digestive dans les premiers jours de vie, d'où la nécessité d'une séparation précoce avec iléostomie ou colostomie (tableau II) [7]. Dans notre observation, la difficulté était due à la méconnaissance du diagnostic au cours de la grossesse, au caractère urgent de la césarienne et de la séparation chirurgicale.

Le pronostic dépend de la nature et de l'extension des organes communs de même que l'association à d'autres malformations [4]. En effet, la majorité des jumeaux conjoints meurent in utéro ou immédiatement après la naissance [2]. De plus, le taux de mortalité atteint 70% en cas de séparation chirurgicale urgente [4]. Le pronostic de notre cas était péjoratif du fait du contexte malformatif auquel se rajoute l'urgence de la séparation chirurgicale.

## Conclusion

L'amélioration de la prise en charge des jumeaux conjoints ne peut se faire qu'en cas de diagnostic prénatal précoce qui permet de préciser les structures anatomiques communes, de rechercher une anomalie congénitale associée, d'organiser l'accouchement dans une structure adaptée et de programmer une prise en charge néonatale multidisciplinaire.
